# Liver‐directed therapies for colorectal liver metastases

**DOI:** 10.1002/cncr.70097

**Published:** 2025-10-10

**Authors:** Andrew D. Folkerts, Lauren M. Janczewski, Ryan P. Merkow

**Affiliations:** ^1^ Department of Surgery Surgical Implementation and Health Science Research Center University of Chicago Chicago Illinois USA; ^2^ Department of Surgery Northwestern University Feinberg School of Medicine Chicago Illinois USA

**Keywords:** colorectal metastases, hepatic ablation, hepatic artery infusion, liver metastases, liver‐directed therapies, multidisciplinary cancer care, transplant oncology

## Abstract

Colorectal cancer is the third most common cancer diagnosed in the United States, with an anticipated 53,000 deaths from this disease in 2025. Greater than one in five patients will present with synchronous colorectal liver metastases (CRLMs), and 25%–30% will develop CRLMs during the course of the disease. In addition, the rate of liver‐specific recurrence is high, with recurrences in 50%–75% of patients who previously underwent resection for liver metastasis. Surgical resection remains the cornerstone of treatment for resectable CRLM, but a significant proportion of CRLMs are unresectable on presentation. Unique treatment strategies have been developed to expand treatment options and improve outcomes. Today, there are multiple established therapies in routine practice as well as novel therapies that are currently under development with promising results to date. Each therapy has its own goals ranging from reducing the chances of locoregional recurrence, supporting an effort to convert unresectable disease burden into potentially resectable tumors, and salvage options with the goal of extending survival. Because most liver‐directed therapies are complementary, it is also important to understand how each option affects other therapies to develop a coordinated treatment strategy. Specifically, it is essential to understand the indications, limitations, and outcomes of each option when making multidisciplinary treatment decisions. In this article, the authors review the current landscape of liver‐directed therapies and briefly discuss promising emerging treatment options.

## Abstract 2

Although resection and ablation remains the preferred therapy for resectable colorectal liver metastasis, a significant proportion of patients with unresectable disease still have many therapy options available. These therapy options, by themselves and in combination, require a multidisciplinary coordinated and tailored strategy that considers a host of factors, including patient characteristics, disease biology treatment goals, and potential complications.

## INTRODUCTION/EPIDEMIOLOGY

Colorectal cancer is the third most common cancer diagnosed in the United States. In 2025, there will be an estimated 154,000 new colorectal cancer cases, and an anticipated 53,000 deaths will occur from this disease.^1^ Surgical resection remains the recommended first‐line treatment for local‐regional disease^2^; however, greater than one in five patients with colorectal cancer will present with synchronous metastases at the time of diagnosis.^3^ Colorectal cancer has an especially high rate of colorectal liver metastasis (CRLM), and a reported 26.5% of patients have CRLM within 5 years of diagnosed localized disease.^4^ In addition, liver‐specific recurrence is high, occurring in 50%–75% of patients who had previously resected liver metastasis.^5^ Surgical resection also remains the cornerstone of treatment for resectable CRLM, but a significant proportion of patients with CRLM are unresectable on presentation, complicating treatment decisions and options.^6^, ^7^, ^8^ Before the advent of liver‐directed therapies, systemic chemotherapy was the only option for unresectable CRLM. Fortunately, over the past 50 years, unique treatment strategies have developed to expand treatment options and improve outcomes. Today, there are multiple established therapies in routine practice as well as novel therapies that are currently under development, with promising results to date. Each therapy has its own goals, ranging from reducing the chances of local recurrence, supporting an effort to convert unresectable disease burden to potentially resectable tumors, and salvage options with the goal of extending survival. Because most liver‐directed therapies are complementary, it is also important to understand how each option affects other therapies such that a coordinated, multidisciplinary treatment strategy can be developed. In this article, we review the current landscape of liver‐directed therapies and briefly discuss promising options.

## IMAGING AND STAGING

The goals of imaging for CRLM are to identify all liver lesions with their corresponding anatomic relations; understand the often variable hepatic arterial, venous, and biliary anatomy; assess for the presence of extrahepatic disease; and estimate liver volumetry, all of which have implications for treatment decisions.^9^ Imaging modalities that are used in CRLM include magnetic resonance imaging (MRI), contrast‐enhanced computed tomography (CT), and positron emission tomography (PET)/CT with sensitivity of 93.1%, 82.1%, and 74.1% respectively, for lesion detection and localization in chemotherapy‐naive patients.^9^, ^10^ Although it has lower sensitivity than MRI, contrast‐enhanced CT is most often used given its ability to rapidly evaluate the chest, abdomen, and pelvis.^11^ However, MRI has an even more pronounced sensitivity advantage over CT for lesions <1 cm, in hepatic steatosis, and after chemotherapy administration.^11^, ^12^, ^13^, ^14^, ^15^ PET/CT is relegated to discovering extrahepatic disease but can be helpful to distinguish active liver disease in certain situations.^11^, ^16^ If the patient undergoes a surgical procedure, intraoperative ultrasound should be used to confirm the findings seen on preoperative imaging.^11^, ^17^

Expert surgeons—despite agreeing on the technical definition of *resectable* as able to remove the disease burden while having adequate vascular inflow, outflow, and biliary drainage with sufficient future liver remnant (FLR)—often disagree on which specific disease burden qualifies as resectable.^18^, ^19^ Agreement on easily resectable and very advanced disease is relatively simple, while the main difficulty is with potentially resectable disease.^19^, ^20^ Alternatively, patients with potentially resectable disease may benefit from procedures aimed at increasing the FLR or from liver‐directed therapies with the goal of downstaging the disease burden.^21^ Procedures that increase the FLR include portal vein embolization, combined portal and hepatic vein embolization, and associating liver partition with or without portal vein ligation for staged hepatectomy.^21^ These procedures induce hypertrophy with variable kinetics; however, 14%–20% of patients may have progression of disease while awaiting hypertrophy.^22^, ^23^ No matter the patient's current disease status, they should have imaging every 2 months while on systemic treatments to evaluate resectability.^20^, ^24^ Understanding resectability as well as the many other factors, ranging from patient comorbidities and disease burden to molecular diagnostics, indications, side effects, and effectiveness of all the available treatment modalities, highlights the need for multidisciplinary management to individualize the treatment strategy for each patient.

## RESECTION AND ABLATION

Among all liver‐directed therapies, *surgical resection* is considered the preferred treatment option if the patient is a surgical candidate.^7^, ^25^, ^26^, ^27^ The goal of surgical resection is complete removal of all disease while leaving behind an adequate FLR with inflow (arterial and portal venous blood vessels), outflow (hepatic veins), and an intact and draining biliary system. Parenchymal‐sparring hepatectomies are preferred over anatomic resections, when possible, given the reduction in surgical morbidity with equivalent oncologic outcomes.^28^, ^29^ In addition, hepatic ablation is an acceptable alternative to resection and should be considered when lesions are small (<3 cm), intraparenchymal, and would not injure critical structures (e.g., proximal inflow pedicles).^30^ Hepatic ablation allows the surgeon to avoid major anatomic resections, thus sparing healthy liver parenchyma to avoid limiting future resection options; this is particularly important for CRLMs, in which recurrences are common.^31^

There are three main methods of liver ablation, including *microwave ablation* (MWA), *irreversible electroporation* (IRE), and *radiofrequency ablation* (RFA). In the United States, MWA is the main method used today, with some select indications for IRE.^32^ MWA uses an antenna, which is inserted through the liver parenchyma to reach the center of the tumor. Intraoperative ultrasound is used to guide the antennae to each visualized lesion. A similar technique utilized by interventional radiology is also an option using a percutaneous approach.^33^ Conversely, IRE uses two probes that bracket either side of the tumor, and an electrical current is passed between the two probes, creating microperforations in the cells.^34^ The microperforations cause cell lysis and apoptosis. To decrease the risk of cardiac arrhythmia, the therapy is synchronized with the heart rhythm to apply the current only during the absolute refractory period.^34^ RFA is less commonly used in the United States for CRLMs because of increased heat sink and less accuracy, but its rationale and use are similar to those for MWA.

### Indications and advantages

Surgical resection is indicated when lesions are resectable, as defined by having an adequate FLR. Anatomic resections (e.g., right hepatectomy) are increasingly uncommon and should be avoided if a parenchymal‐sparing approach (e.g., wedge resection, segmentectomy) is feasible while achieving negative margins.^35^, ^36^ In cases of smaller intraparenchymal lesions, difficult anatomic locations (e.g., near the junctions of the hepatic veins and inferior vena cava), and in patients who are poor operative candidates secondary to comorbidities, MWA is generally the preferred treatment modality.^30^, ^37^ IRE has the additional advantage over MWA in that it can be safely used in close proximity to vascular and biliary structures. Cells in the vessel matrix that are treated by IRE are replaced with new mesenchymal cells while leaving the vessel matrix unharmed.^38^

### Limitations and complications

The main factors influencing the use of resection versus ablation include the number and size of the lesions, their location, and the clinical status of the patient. Resection is primarily used for superficial lesions and in tumors >3 cm when an adequate FLR is predicted.^39^ We primarily use ablation for intraparenchymal lesions <3 cm when remote from proximal inflow pedicles to avoid injury, especially to the biliary system.^40^ In addition, patients with *RAS* mutations have a higher rate of R1 resection and recurrence after ablation and may require wider margins to achieve an R0 resection.^41^, ^42^ Complications of surgical resection include major bleeding, bile duct injury, bile leak, wound infections, and posthepatectomy liver failure if the FLR is inadequate or compromised.^43^ Ablation is generally considered safer than surgical resection but can still lead to injury to surrounding biliary and vascular structures as well as liver abscesses.^44^ Because IRE uses an electrical current, it can induce cardiac arrhythmias and subsequent hemodynamic instability, so patients with a history of ventricular arrhythmias, pacemakers, and implantable cardiac devices may not be candidates.^34^ It is also contraindicated for patients with a history of seizures, although there are currently no documented cases of seizures being caused by IRE.^38^

### Outcomes

Surgical resection remains the gold‐standard, curative‐intent treatment modality with which other modalities should be compared. A study from Memorial Sloan Kettering Cancer Center reported a disease‐specific survival of 4.9 years and a 10‐year overall survival rate of 24% among 1211 patients with CRLMs who underwent hepatectomy.^27^ Although 348 of those patients (28.7%) had hepatic artery infusion (HAI) pump chemotherapy and an unspecified number underwent some form of ablation, this demonstrates the high rate of long‐term survival if complete resection of all liver disease is achieved.

**FIGURE 1:**
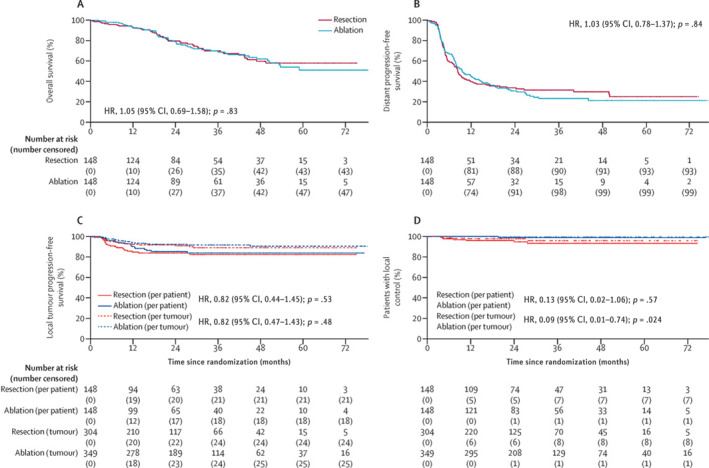
COLLISION randomized trial results comparing hepatic ablation versus surgical resection demonstrating (A) overall survival, (B) distant progression‐free survival, (C) local tumor progression‐free survival, and (D) local control with Cox regression values. CI indicates confidence interval; HR, hazard ratio. Reproduced with permission from van der Lei et al., 2025.^47^

Recent data suggest similar overall survival and local control with ablation versus surgical resection in carefully selected patients. Results from a nonrandomized, single‐institution trial in 2008 comparing RFA with surgical resection indicate that actuarial survival after diagnosis was 40 months for RFA and 59 months for surgical resection. The median disease‐free survival was 9 months for RFA and 30 months for surgical resection, clearly favoring surgical resection for disease‐specific outcomes.^45^ However, it has been demonstrated that MWA produces superior results compared with RFA and has replaced RFA as the ablation method of choice.^37^, ^46^ The recent COLLISION trial (ClinicalTrials.gov identifier NCT03088150) had a total of 300 patients who were randomized to either surgical resection or thermal ablation, defined as either MWA or RFA. Of the 300 patients, 134 underwent MWA, and 11 underwent RFA with the goal of demonstrating the noninferiority of ablation for resectable CRLM. After a median follow‐up of 28.9 months, the trial indicated no difference in overall survival, with 92.7% 1‐year, 78.5% 2‐year, and 51.2% 5‐year overall survival rates for ablation versus 92.9% 1‐year, 79.6% 2‐year, and 58% 5‐year overall survival rates for surgical resection, and with no differences in progression‐free survival, with a median progression‐free survival of 9.6 months for ablation and 8.4 months for resection (Figure 1).^47^ However, there was a significant reduction in low‐grade, high‐grade, and overall adverse events for ablation.^43^, ^44^, ^47^ These results indicate that, in appropriately selected patients, ablation is an equivalent treatment modality to surgical resection.

Importantly, most liver‐directed therapies are complementary and often are used together, especially resection and ablation. This combination occurs so frequently that, in a recent study by our group, 75% of patients underwent concurrent resection and ablation.^48^ In one retrospective, propensity‐matched study, 192 patients with resectable CRLM were treated either with hepatectomy alone or with a combination of hepatectomy and RFA ablation. After matching, more patients were treated with preoperative chemotherapy in the combination group than in the hepatectomy alone group. There was no difference in disease‐free survival in the combination group versus the hepatectomy alone group (9 vs. 10 months, respectively; *p* = .257), intrahepatic recurrence‐free survival (10 vs. 11 months, respectively; *p* = .329), or overall survival (median not reached after 60 months of follow‐up vs. 47 months, respectively; *p* = .358). However, there was a significant decrease in the rate of postoperative hepatic insufficiency (0.0% vs. 5.2%; *p* = .023) and postoperative hospital stay (7 vs. 8 days; *p* = .019) in the combined therapy group,^49^ likely related to an increased extent and complexity of disease. Resection and ablation individually have attained impressive local control, disease‐specific survival, and overall survival for managing CRLM, but they are especially useful and effective when used in a complementary manner.

## HEPATIC ARTERY INFUSION PUMP

HAI pump chemotherapy uses the different blood supply for normal liver parenchyma compared with CRLMs.^50^ Normal liver parenchyma derives its main blood supply from the portal vein and secondarily from the hepatic artery, whereas the main blood supply to CRLMs is the hepatic artery. Therefore, chemotherapy is supplied through the hepatic artery, targeting the CRLMs while limiting toxicity to normal liver parenchyma.^51^

The procedure involves dissection of the common hepatic, proper hepatic, and gastroduodenal artery with a limited portal lymphadenectomy. The catheter is most often placed in the gastroduodenal artery.^52^ To prevent the prohibitively high concentrations of chemotherapy from having off‐target effects outside of the liver, blue dye is injected to visualize the perfusion of the HAI, and ligation of side branches is performed.^53^ Cholecystectomy is also performed to prevent chemotherapy‐induced cholecystitis, and the pump is placed in the subcutaneous tissues. Heparinized saline is loaded in the pump to prevent catheter occlusion, and chemotherapy administration is started 2 weeks from HAI pump placement.^52^

### Indications and advantages

HAI has two primary indications for CRLMs. It can be used in the adjuvant setting after all liver disease is addressed with the primary goal of decreasing hepatic recurrence, which occurs in >50% of patients.^5^ HAI therapy can also be used as part of a multistage strategy in an attempt to convert patients who have unresectable CRLM to completely resectable status.^54^ Another strategy currently being evaluated as part of a multi‐institutional, randomized trial is using HAI as an adjunct to systemic chemotherapy among patients who are permanently unresectable.^55^, ^56^

The infusion through the hepatic artery, and not through the branches, allows for treatment of the entire liver, making it a treatment choice for multiple metastasis in different regions of the liver.^52^ In addition, the chemotherapy agent most often used for HAI, floxuridine (FUDR), has a clinically complete first‐pass metabolism, which allows for significantly higher concentrations of chemotherapy in the liver while limiting systemic toxicity.^51^, ^57^

### Limitations and complications

Because near complete first‐pass metabolism of FUDR occurs, HAI therapy is generally always given with concurrent systemic chemotherapy to control micrometastatic extrahepatic disease and the primary tumor if it is still in place.^58^, ^59^ Nevertheless, systemic therapy is dose‐reduced by approximately 20% and may affect the development or control of extrahepatic disease. Patients with *BRAF* mutations have an elevated risk of extrahepatic disease, a decreased response to chemotherapy, and an increased risk of recurrence; therefore, these patients are often excluded from HAI, although exceptions do exist if the biology of disease has proven otherwise favorable.^60^, ^61^ In addition, aberrant hepatic vascular arterial anatomy in some patients limits the use of HAI or potential future liver‐directed surgical options.^52^

Complications of the HAI pump are fortunately low when performed by an experienced team^62^; however, potential complications include arterial pseudoaneurysm, hepatic artery thrombosis/dissection, hematoma, catheter dislodgement resulting in hemorrhage, infection of the pump, and pump migration.^48^, ^63^, ^64^ One of the most serious complications is biliary sclerosis secondary to FUDR toxicity to the biliary system.^65^ Fortunately, this is uncommon, does not appear to affect overall survival, and can usually be managed with biliary stenting procedures.^48^ Furthermore, alternative dosing regimens have shown promise in further reducing this dreaded complication. Finally, an important, potentially life‐threatening complication that may occur if not recognized is extrahepatic perfusion of chemotherapy, causing off‐target toxicities such as peptic/duodenal ulcers and hemorrhage.^64^

### Outcomes

**FIGURE 2:**
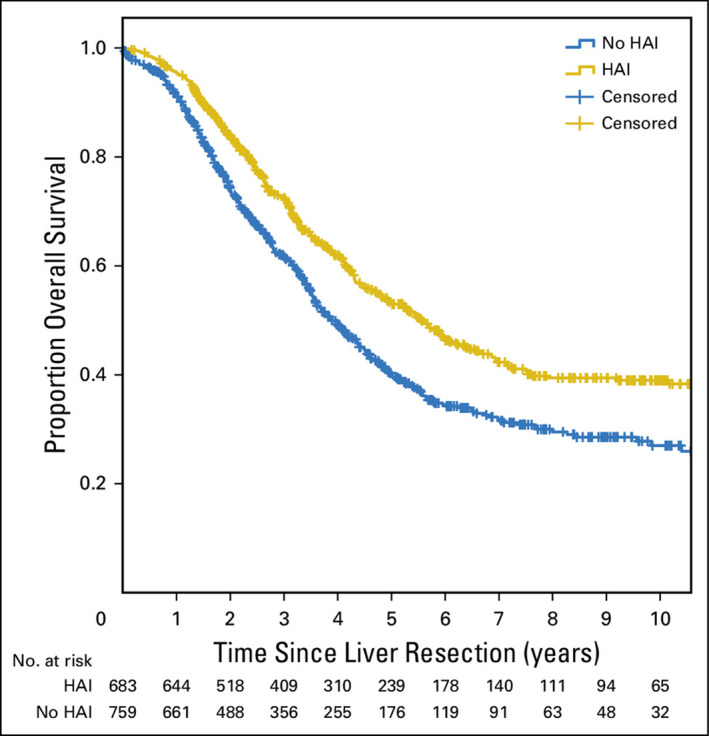
Overall survival among patients receiving adjuvant hepatic artery infusion pump chemotherapy versus modern systemic chemotherapy. HAI indicates hepatic artery infusion. Reproduced with permission from Groot Koerkamp et al., 2017.^68^

HAI has demonstrated significant utility in the adjuvant setting.^66^, ^67^ In the landmark phase 3 trial for HAI in the adjuvant setting, which included 156 patients, Kemeny et al., observed that the addition of HAI improved the primary outcome, progression‐free survival, from 17.2 to 31.3 months, and 10‐year overall survival rates were 41.1% in the combined therapy group versus 27.2% in the patients who received systemic chemotherapy alone.^66^, ^67^ In the largest adjuvant series, which included 2368 patients treated at Memorial Sloan Kettering Cancer Center from 1992 to 2012, Groot Koerkamp et al. observed an improved median overall survival of 67 months for patients who received HAI (10‐year overall survival, 38%) compared with a median overall survival of 44 months in those who did not receive HAI (10‐year overall survival, 24%; Figure 2).^68^ Of note, patients in the HAI group had more advanced disease than those who did not receive HAI. It is worth noting that there was an even more significant survival benefit for patients who had lower Fong scores with HAI (89 months with HAI vs. 53 months without HAI).^69^ Finally, the survival benefit with HAI was even more pronounced in lymph node‐negative patients, with a median overall survival of 129 months in those who received HAI versus 51 months in those who did not.^68^ HAI has extensive data demonstrating its effectiveness in multiple stages of disease, and providers should consider referral to HAI centers for potentially eligible patients.

In the unresectable setting, conversion rates to complete resection vary widely, depending on patient selection, and range between approximately 10% and 50% with higher rates of conversion associated with patients who are chemotherapy‐naive.^48^, ^70^, ^71^, ^72^, ^73^ Pak et al. observed that 33 of 64 patients (52%) who received HAI had a conversion to resectable disease at a median follow‐up of 81 months.^73^ Importantly, the only variable associated with improved overall survival in that study was conversion to resection.^73^ Our group, in a series of patients outside of Memorial Sloan Kettering Cancer Center, reported a conversion rate of >30%.^51^ Some data exists indicating a survival benefit of HAI in the unresectable setting regardless of conversion to resection. In another landmark study by Kemeny et al., HAI vs. systemic therapy alone was associated with a significant improvement in overall survival of 24.4 months versus 20 months, including improvements in objective response rates, the time to hepatic progression, and quality of life.^58^ This improvement was also reported in chemotherapy‐refractory patients, with one retrospective study demonstrating a 29% partial response rate, a 50% rate of stable disease, and a median overall survival of 17.2 months with HAI treatment after progression with up to three chemotherapy regimens.^74^ The PUMP trial (ClinicalTrials.gov identifier NCT05863195) is a multi‐institutional, randomized controlled trial currently evaluating whether the addition of HAI to systemic chemotherapy alone in patients with permanently unresectable CRLMs improves overall survival and is currently enrolling patients.^55^

## TRANSARTERIAL RADIOEMBOLIZATION

Transarterial radioembolization (TARE), like HAI, uses the fact that CRLMs receive arterial inflow only, whereas normal liver parenchyma receives both portal venous and arterial inflow.^53^ TARE also uses the radiosensitivity of the tumor by delivering targeted internal radiation therapy, most often using yttrium‐90 beads, through the hepatic artery.^75^ Before the administration of TARE, extensive vascular imaging is used to reveal additional arteries that may lead to extrahepatic treatment and side branches that may be embolized.^76^ In particular, the lung‐shunting fraction determines the risk of radiation pneumonitis.^75^

### Indications and advantages

The goal of this treatment is for local control and is not generally curative.^77^ Depending on the number of tumors and their relative location to each other, yttrium‐90 beads can be administered in a regional or local fashion.^76^ The Radioembolization Brachytherapy Oncology Consortium recommends limiting TARE to patients who have a life expectancy of at least 3 months, minimal side branches to other gastrointestinal organs, minimal pretreatment liver parenchymal disease, and an acceptable risk of off‐target radiation delivery.^78^

### Limitations and complications

This therapeutic option is usually not used with curative intent, as stated above, and thus is uncommonly chosen as a first‐line therapy. In addition, when extensive radiation doses are used in the liver, other modalities are no longer appropriate, namely, HAI chemotherapy. This is primarily because of concerns regarding liver injury.^79^ In cases of super‐selective treatment to an individual tumor or liver segment, HAI can still be carefully considered and potentially employed. Therefore, the order of these therapies should be coordinated.

Common complications of this procedure include abdominal pain, nausea, vomiting, lymphopenia, and elevation of liver function tests and bilirubin.^76^ More serious complications can include liver toxicity causing dysfunction, including liver failure, biliary complications like cholecystitis, and injury to off‐target organs.^80^

### Outcomes

**FIGURE 3:**
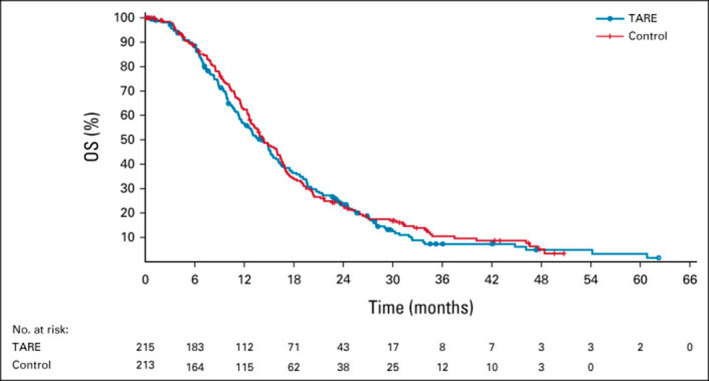
Randomized trial overall survival results comparing trans‐arterial radioembolization vs. second‐line chemotherapy alone among patients with colorectal liver metastases. OS indicates overall survival; TARE, transarterial radioembolization. Reproduced with permission from Mulcahey et al., 2021.^82^

A prospective, multicenter, phase 2 study with 151 patients who had liver metastases, which included 61 patients who had CRLM, with refractory disease underwent TARE.^76^ The median progression‐free survival was 2.9 months, and the median overall survival was 8.8 months for the patients with CRLM, which were improvements from the reported 4‐month to 6‐month overall survival for the patients who had refractory disease.^81^ A randomized, multinational phase 3 trial also was completed for those who had progression on first‐line chemotherapy and were treated with either second‐line chemotherapy alone or with TARE. The study included 428 patients from 95 centers and reported a hepatic progression‐free survival of 9.1 months in the TARE group versus 7.2 months in the chemotherapy‐alone group. There was also a significant increase in progression‐free survival of 8.0 months in the TARE group compared to 7.2 months with chemotherapy alone. However, importantly, there was no difference in overall survival between the 2 groups (14.0 months for TARE vs. 14.4 months for chemotherapy alone; Figure 3).^82^ Given this very small increase in progression‐free survival and failure to demonstrate any significant increase in overall survival, the use of TARE to primarily treat CRLMs should be limited to selected patients as part of a broader treatment strategy. Because TARE is often used in combination with other liver‐directed therapies, often in the palliative setting, it should be discussed in a multidisciplinary setting in centers of excellence with significant experience.

## EXTERNAL RADIATION

External radiation exploits the radiosensitivity of CRLM, most often through stereotactic body radiotherapy (SBRT), with the goal of local control.^83^ For SBRT, patients are placed in a thermoplastic body mask with a Styrofoam block to decrease breath movements while radiation is applied using a linear accelerator or a cyberknife.^84^ Although studies vary on the exact dosage of radiation, somewhere between 60 and 180 grays (Gy) of radiation is supplied with roughly 30−60 Gy per fraction.^83^ Of note, the dosage of SBRT for liver metastasis was one of the only topics for which an expert panel was not able to reach consensus.^85^ An MRI linear accelerator (MRI LINAC) uses a four‐dimensional MRI to target lesions through the breath cycle and thus is even more precise than SBRT. Both of these techniques are more precise than traditional radiation therapy, which decreases radiation toxicity to normal tissue.^86^

### Indications and advantages

External radiation is used primarily when there is inadequate liver reserve to undergo surgical resection or when the patient is not able to undergo surgical resection because of other comorbidities that would make surgical intervention prohibitively dangerous.^83^ It is also used when the usually preferred ablation is not available or feasible.

Advantages include that external radiation is not as limited by close proximity to critical anatomic structures, such as blood vessels or the diaphragm, and it does not have the same size limitation as ablation, although 4 cm is often cited as the maximum greatest dimension most effectively treated with external radiation.^84^ In contrast to Histotripsy (discuss later), which is also noninvasive, SBRT only requires the immobilization of the body mask and does not need procedural sedation. Finally, because of its four‐dimensional imaging technique, MRI LINAC has the additional advantage that it is able to change the radiation plan during the procedure allowing for more individualized treatment. This allows for a more targeted and high‐intensity *ablative* dose to liver tumors.^86^

### Limitations and complications

Although local control is often able to be achieved in appropriately selected patients, the dose needed to definitively treat adenocarcinoma is 70 Gy, whereas the liver lifetime dose maximum is 35 Gy, necessitating high precision, for example, with MRI LINAC.^87^, ^88^ However, this high precision comes at a high resource cost, with few centers investing in the infrastructure needed for MRI LINAC.^89^

Although SBRT and MRI LINAC are relatively precise in their targeting, radiation side effects occur. Symptoms of radiation effects to the normal liver parenchyma include liver toxicity, most often presenting with an asymptomatic rise in liver enzyme levels, although very rarely it can be as severe as liver failure.^83^ Other symptoms include nausea and fatigue, which are experienced quite frequently, and biliary stenosis, which is uncommon.^90^ In addition, the long‐term effects of radiation make any future therapy, particularly surgery, more challenging and, in some cases, not possible.

### Outcomes

One recent phase 2 prospective trial included 61 patients with unresectable liver oligometastatic CRLMs, of whom 29 received SBRT. Most (82%) received 75 Gy in three daily fractions of 25 Gy and were followed for 5 years. Local control rates at 1, 3, and 5 years were 94%, 78%, and 78%, respectively. However overall survival rates at 1, 3, and 5 years were 85%, 31%, and 18%, respectively, and 94% of the patients who died did so from disease progression.^84^ One systematic review of SBRT trials for CRLM indicated that the pooled 1‐year and 2‐year rates of local control were 67% and 59%, respectively; and respective the overall survival rates at 1 and 2 years were 67% and 57%.^83^ Additional research with phase 3 trials is needed to determine the optimum radiation dosage and patient selection criteria as precision increases and off‐target effects decrease. Similar to TARE, external radiation is often combined with other local therapies and should be considered by expert multidisciplinary teams.

## TRANSPLANTATION

Liver transplantation has become a standard of care for hepatocellular cancer and cholangiocarcinoma.^91^ However, early studies of liver transplantation for CRLM revealed a 5‐year survival rate of only 18%.^56^ Considering the much higher survival rate for other indications and the scarce number of livers available, CRLM was considered a contraindication for transplantation.^56^, ^91^ Recent research, including observational data at experienced international centers and the TRANSMET trial (ClinicalTrials.gov identifier NCT02597348), challenged these earlier results with re‐inclusion of CRLM as an indication for transplantation in highly selected patients.^92^, ^93^ The method of liver transplantation has been detailed extensively elsewhere and is not significantly different than that of organ transplantation for other indications.^94^

### Indications and advantages

The goal of this treatment is complete removal of the entire cancer burden en bloc and replacement with a cancer‐free organ. This complete removal of the cancer burden can only be completed if the liver is the only source of metastatic burden. The TRANSMET study, published in *The Lancet* in 2024, compared patient outcomes between chemotherapy alone versus chemotherapy and liver transplantation. ^93^ Patients included in this trial had permanently unresectable CRLM, underwent standard oncologic resection of the primary with no local recurrence on colonoscopy, received neoadjuvant chemotherapy with a partial response or stable burden of disease for 3 months on three or less chemotherapy agents, had an Eastern Cooperative Oncology Group performance status of 0 or 1, were younger than 65 years, had no *BRAF* mutation, and had a carcinoembryonic antigen level <80 μg/L or a 50% reduction with systemic treatment. Contraindications to transplantation were similar to those to transplantation for other indications and included active substance abuse, lack of support, other malignancies, and no health insurance. ^93^ In addition, an international consensus panel that was completed before the TRANSMET trial started made 44 statements regarding transplantation for patients with CRLM that included considering patients who had unresectable CRLM for transplantation if they had 6 months of stability or 1 year of response while still remaining unresectable on bridging therapy.^95^ Patients were recommended to be excluded from transplantation if they had resectable disease, poorly differentiated adenocarcinoma, signet ring pathology, *BRAF V600E* mutations, progression (determined either radiographically or biochemically) on chemotherapy with relative exclusion if they had N2 or higher disease, *RAS* mutations, high microsatellite instability or mismatch‐repair deficiency, and carcinoembryonic antigen level >80 μg/L with an increasing trend, among others.^95^ It is important to note that other liver‐directed therapies, namely HAI, were not a contraindication to transplantation.

### Limitations and complications

As detailed above, the inclusion criteria to be considered for liver transplantation are very extensive, significantly limiting the number of patients who are eligible to receive a transplant.^93^

Liver transplantation has many complications relating to the surgical procedure, including organ rejection and immunosuppression. In the acute setting, the common causes of mortality include technical issues with hemorrhage, sepsis, and organ primary nonfunction. In the long‐term setting, common causes of mortality, in the order from most to least common, include organ rejection with liver failure, malignancy, cardiovascular, infection, and kidney failure.^96^ In the TRANSMET population, complications included biliary complications, pulmonary complications, allograft dysfunction, primary nonfunction, hemorrhage, and surgical site infection.^93^

### Outcomes

**FIGURE 4:**
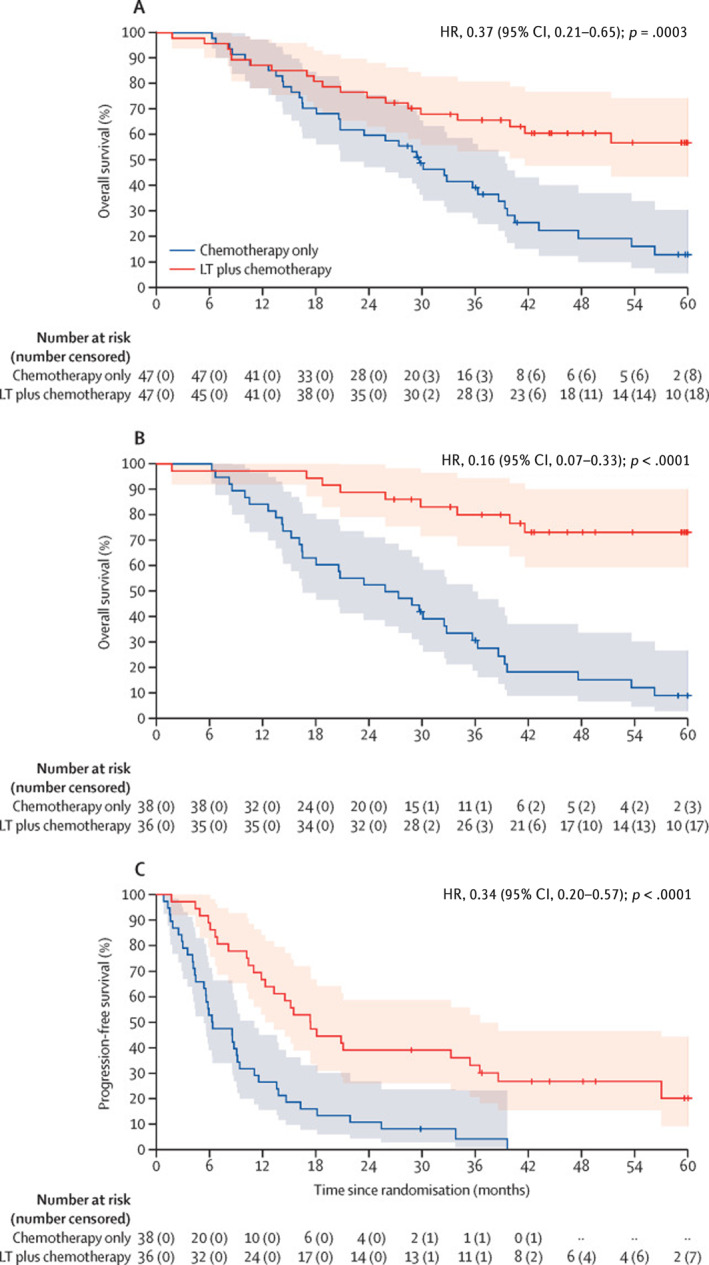
TRANSMET trial results comparing liver transplantation plus chemotherapy versus chemotherapy alone demonstrating (A) overall survival in the intention‐to‐treat population, (B) overall survival in the per‐protocol population, and (C) progression‐free survival in the per‐protocol population. CI indicates confidence interval; LT, liver transplantation. Reproduced with permission from Adam et al., 2024.^93^

The TRANSMET study included 47 patients randomized to the liver transplantation group, of whom 38 ended up undergoing liver transplantation. In the intention‐to‐treat protocol, the chemotherapy‐alone group had a 5‐year overall survival rate of 12.6%, whereas the chemotherapy plus transplantation group had a 5‐year overall survival rate of 56.6%, with an estimated restricted mean survival time of 31.3 months and 43.9 months, respectively.^93^ The per‐protocol analysis revealed a 5‐year overall survival rate of 9.3% for the chemotherapy‐alone group and 73.2% for chemotherapy plus liver transplantation group. The median progression‐free survival was 6.4 months in the chemotherapy‐alone group and 17.4 months in the transplantation group, with 5‐year progression‐free survival rates of 0% and 19.9%, respectively (Figure 4).^93^ Given the extensive inclusion and exclusion criteria of the TRANSMET trial, further studies are needed to help guide improved patient selection criteria to better understand how transplantation fits into the algorithm of other liver‐directed therapies

## NEW AND EMERGING TECHNOLOGIES: HISTOTRIPSY

The name *histotripsy* was coined in 2004 and comes from the combination of *histo*, meaning soft tissue, and *tripsy*, meaning to breakdown.^97^ The treatment uses directed, high‐amplitude, short‐duration, and low‐duty ultrasound with the purpose of applying high negative pressure for the creation of cavitation bubbles that, when they collapse, cause cellular lysis.^97^, ^98^

The procedure starts with the initiation of general anesthesia. Using two ultrasound probes, one diagnostic and one therapeutic, attached to a fully automated arm, the probes are submerged in cooled water, and any gas is removed. The automated treatment is then applied to the predetermined area from preprocedural cross‐sectional imaging. Preprocedural cross‐sectional imaging guides the automated ultrasound probes into place.^99^

### Indications and advantages

Because this technique is noninvasive, it is potentially an option for individuals who are at high operative risk. The cavitation method of cell lysis has minimal spread to nearby structures, creating a relatively exact boundary of treatment.^97^, ^98^ In addition, the creation of cavitation bubbles relies on the tensile strength of tissues. Therefore, nearby structures that have a higher tensile strength than the tumors, such as large blood or biliary vessels, are not compromised by the treatment.^97^, ^98^ The immediate change in ultrasound appearance of treated versus untreated tumor allows for real‐time verification of treatment success.^98^ Repeat imaging can also be done within 1 or 2 months for short‐interval retreatment.^97^, ^98^ Finally, the lysis of the cells creates an exposure of nondenatured cancer antigens, which can possibly provide an abscopal effect to activate the immune system against other, nontargeted tumors.^97^, ^98^

### Limitations and complications

Because this therapy depends on the external application of ultrasound, tumors that are far from the skin surface (e.g., lesions deep in the liver or in patients with a body mass index >30 kg/m^2^), may be hard to visualize and treat.^98^ In addition, the procedure currently requires general anesthesia as well as paralysis, which is not preferable in high‐risk patients.^97^, ^99^ Moreover, the technique usually is limited to treating one to three lesions at a time and thus may require repeat procedures to adequately address all disease.^100^

Histotripsy has had minimal adverse events reported at this time, with asymptomatic elevation of liver enzymes, a perfusion defect, and a subclinical portal vein thrombosis reported in the THERESA study (ClinicalTrials.gov identifier NCT03741088).^99^ A total rate of 7% grade ≥3 complications was reported in initial data from the #HOPE4LIVER trial (ClinicalTrials.gov identifiers NCT04572633 and NCT04573881) without specification of the complication types.^100^, ^101^ Although histotripsy is relatively precise, air‐filled structures between the skin and the treatment area are at risk of cavitation, leading to off‐target damage.^98^

### Outcomes

Most of the current studies on histotripsy have been on animals, and human trial information is still relatively sparse. However, one of the human trials, THERESA, was a single‐institution trial that included eight patients who had liver malignancies, including five with CRLM, and reported tumor destruction, as shown on MRI 1 day after treatment in all of the treated tumors, with an abscopal effect noted in two patients.^99^ In addition, the #HOPE4LIVER trial is a multicenter, nonrandomized, prospective trial between Europe and the United States that is enrolling patients with HCC or liver metastasis who have not responded to, were intolerant to, or had a relapse after other therapies.^100^ These patients are undergoing histotripsy with the goal of demonstrating technical success, as defined by postprocedural imaging, and procedural safety.^100^ The study is ongoing but initial results of the first 44 patients indicated successful treatment in 96% of tumors with subsequent US Food and Drug Administration approval of histotripsy for liver tumors.^101^ Currently, there are no published results on progression‐free survival or overall survival with histotripsy, but 5‐year results of the #HOPE4LIVER trial are anticipated in 2026.

## Conclusion

CRLM remains a complex disease requiring a multidisciplinary approach and consideration of a range of treatment options. Although resection remains the preferred therapy for those who are eligible, significant proportions of patients with unresectable disease still have many options available. The appropriate choice and combination of therapy options require a personalized approach that considers a host of patient factors, preferences, as well as therapy options, indications, goals of therapy, and potential complications, as detailed above.

## AUTHOR CONTRIBUTIONS

**Andrew D. Folkerts**: Writing—original draft; visualization; investigation. **Lauren M. Janczewski**: Writing—review and editing. **Ryan P. Merkow**: Supervision; conceptualization; writing—review and editing.

## CONFLICT OF INTEREST STATEMENT

The authors declare no conflicts of interest.

## Data Availability

The authors have nothing to report.
